# Cryo-Gel embedding compound for renal biopsy biobanking

**DOI:** 10.1038/s41598-019-51962-8

**Published:** 2019-10-24

**Authors:** Malou L. H. Snijders, Marina Zajec, Laurens A. J. Walter, Remco M. A. A. de Louw, Monique H. A. Oomen, Shazia Arshad, Thierry P. P. van den Bosch, Lennard J. M. Dekker, Michail Doukas, Theo M. Luider, Peter H. J. Riegman, Folkert J. van Kemenade, Marian C. Clahsen-van Groningen

**Affiliations:** 1 Department of Pathology, Erasmus MC, Rotterdam, The Netherlands; 2 Department of Neurology, Erasmus MC, Rotterdam, The Netherlands; 3 Department of Clinical Chemistry, Erasmus MC, Rotterdam, The Netherlands

**Keywords:** Mass spectrometry, Kidney diseases

## Abstract

Optimal preservation and biobanking of renal tissue is vital for good diagnostics and subsequent research. Optimal cutting temperature (OCT) compound is a commonly used embedding medium for freezing tissue samples. However, due to interfering polymers in OCT, analysis as mass spectrometry (MS) is difficult. We investigated if the replacement of OCT with Cryo-Gel as embedding compound for renal biopsies would enable proteomics and not disturb other common techniques used in tissue diagnostics and research. For the present study, fresh renal samples were snap-frozen using Cryo-Gel, OCT and without embedding compound and evaluated using different techniques. In addition, tissue samples from normal spleen, skin, liver and colon were analyzed. Cryo-Gel embedded tissues showed good morphological preservation and no interference in immunohistochemical or immunofluorescent investigations. The quality of extracted RNA and DNA was good. The number of proteins identified using MS was similar between Cryo-Gel embedded samples, samples without embedding compound and OCT embedded samples. However, polymers in the OCT disturbed the signal in the MS, while this was not observed in the Cryo-Gel embedded samples. We conclude that embedding of renal biopsies in Cryo-Gel is an excellent and preferable alternative for OCT compound for both diagnostic and research purposes, especially in those cases where proteomic analysis might be necessary.

## Introduction

A renal biopsy is often necessary to make a diagnosis in various disease settings affecting both native and transplant kidneys. Biobanking of clinically indicated kidney biopsies is an optimal reservoir for research purposes which is of great importance in understanding the underlying pathophysiology in many kidney diseases^1,2^. For future studies it is vital that renal tissue is stored in a fashion that maximizes tissue preservation which is crucial for the quality of RNA, DNA and protein retrieval, without interfering with diagnostic evaluation.

Proteomic analysis by mass spectrometry (MS) has been proven to be a powerful tool in the diagnosis and investigation of many kidney diseases^3^. MS is an analytical technique for protein assessment to identify and quantitate molecules based on their mass-to-charge of gas-phase ions. With the use of MS direct analysis of complete sets of proteins in a given tissue sample could be obtained^4^. This could help us in the identification of disease specific proteins in kidney tissue and to better understand the pathogenesis of kidney diseases. For example, MS is used as an ancillary tool for typing of amyloidosis and is used to confirm and identify immunoglobulins in immune-complex mediated proliferative glomerulonephritis and complement factors in complement mediated proliferative glomerulonephritis^5,6^. Furthermore, the finding of disease specific proteins could lead to diagnostic and prognostic biomarkers for disease diagnosis and new therapeutic interventions^7,8^.

Renal biopsies are often either fixed in formalin and then embedded in paraffin or fresh snap frozen. It is a challenge to perform proteomic analysis on formalin-fixed paraffin-embedded (FFPE) tissue due to the formation of both intra- and intercellular crosslinking between proteins^9–11^. MS analyses perform best on proteins extracted from fresh snap frozen tissue. Since frozen tissue without embedding compound is difficult to cut an embedding medium is often used.

Currently the most common medium in which biopsy material is snap frozen is optimal cutting temperature (OCT) compound, a cryopreservative medium composed of polyethylene glycol (PEG), polyvinyl alcohol (PVA) and nonreactive ingredients^12^. OCT stabilizes tissue allowing easy positioning of tissue samples in the microtome. Since the consistency of frozen OCT is more or less the same as the frozen tissue sample and since OCT provides a smooth cutting surface, the quality of sectioning is good. Furthermore, OCT is effective in preserving morphologic and immunohistochemical characteristics^4,12^.

Unfortunately, OCT medium also has disadvantages. MS analyses of OCT embedded tissue is difficult due to the presence of water soluble synthetic polymers. The interference of these polymers in the MS analysis causes suppression of ion formation^9^. In addition, the presence of high polymer peaks of OCT in the mass spectra may hide other smaller peaks^13^. It is therefore vital that OCT is removed from samples before MS analysis is performed which is a complex and time-consuming procedure resulting in a lower protein yield^9,12,14,15^.

Cryo-Gel, a possible alternative embedding medium for OCT, is a highly viscous, biodegradable and completely water-soluble medium. Cryogels are polymeric gels formed after freezing of the solvent (most often water). They are known to be of significant interest in various areas and are often used in tissue engineering and biotechnology^16,17^. In the current study we investigate the feasibility of Cryo-Gel for snap freezing of renal biopsies in routine diagnostics and subsequent research analysis. In addition, Cryo-Gel embedded tissue samples from normal spleen, skin, liver and colon were analyzed.

## Results

**Figure 1:**
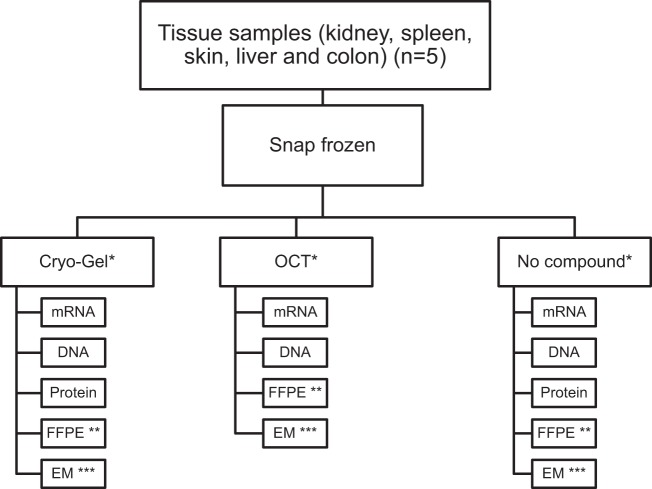
Flowchart showing the work-up protocol performed on normal tissue samples from kidney, spleen, skin, colon and liver. Samples were embedded in either Cryo-Gel, OCT or without compound. *Two samples were embedded from the different tissues for each embedding medium. For the renal tissues, the additional performed techniques were all performed in duplicate. **H&E was performed on all tissue samples. PAS, Jones, Trichrome and immunohistochemistry for AE1/AE3 and CD31 were performed in the renal samples. PAS, trichrome, Sirius Red and Iron stain were performed in the liver samples. ***EM was only performed on the renal samples.

The work-up protocol was performed on normal tissue samples embedded in either Cryo-Gel, OCT or without compound (Fig. 1). Sections of both Cryo-Gel and OCT embedded samples were easily cut. However, samples snap-frozen without embedding compound were less stable and therefore less easily cut.

### Histology, histochemistry and immunohistochemistry

**Figure 2:**
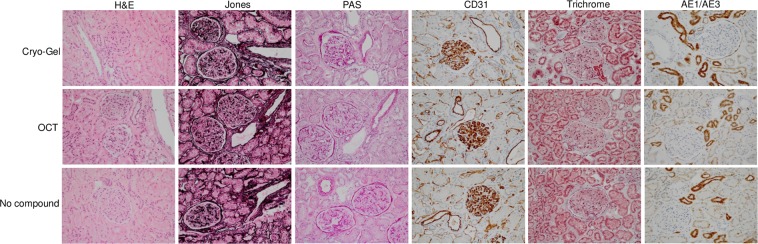
H&E, Jones, PAS, CD31, Trichrome and AE1/AE3 staining on frozen renal tissue samples embedded in Cryo-Gel, OCT and without compound converted to FFPE material (magnification 20x).

Tissue morphology of hematoxylin and eosin (H&E) stained frozen sections and histochemistry and immunohistochemistry on sections of frozen samples converted to FFPE material showed good results in all methods of embedding (Fig. 2 and Supplementary Fig. 1). No differences in background staining were observed. However, OCT embedded tissue showed more eosinophilic cytoplasm in the liver and spleen tissue samples and the nuclei were darker in the renal tissue samples in the H&E staining.

### RNA

**Figure 3:**
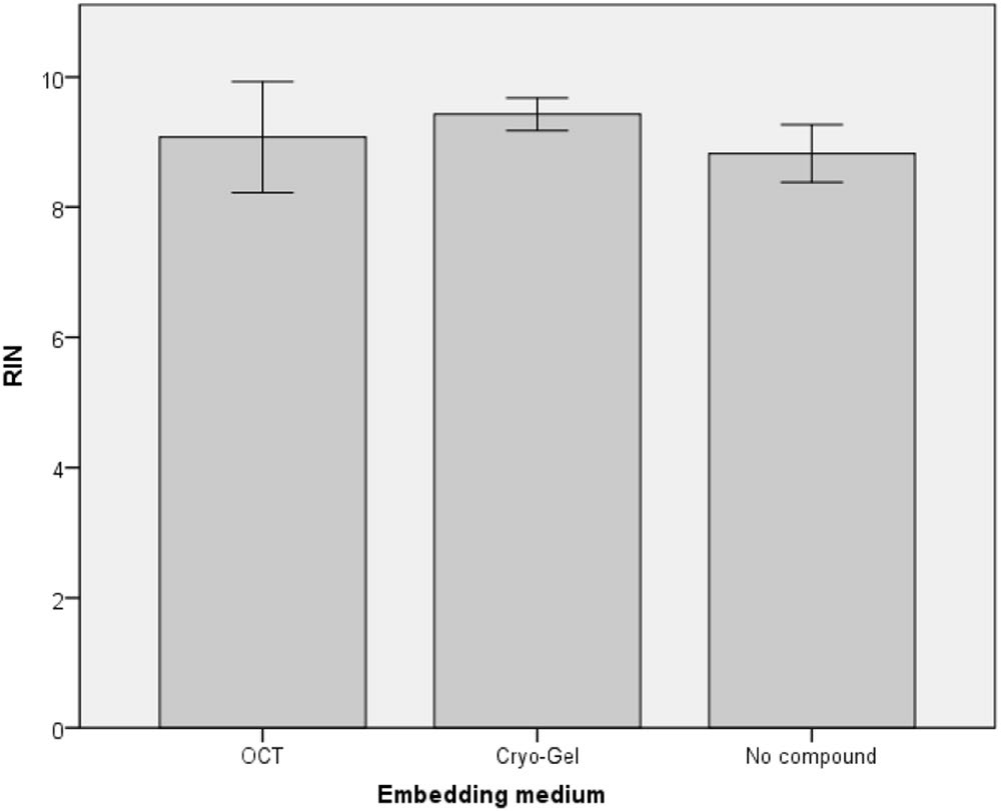
Mean RIN values for samples embedded in Cryo-Gel, OCT and without compound.

RNA analysis was performed in duplicate on both renal samples for each embedding method. In the renal tissues, the Cryo-Gel samples showed a mean RNA concentration of 289.8 ng/µl. In the OCT embedded samples and the samples without compound the mean RNA concentration was 210.0 ng/µl and 192.5 ng/µl respectively. No significant differences between RNA concentrations were measured between the different embedding methods (p = 0.094) in the renal samples. A mean RNA Integrity Number (RIN) of 9.43 ± 0.13, 9.08 ± 0.43 and 8.83 ± 0.22 was found for the renal samples embedded in Cryo-Gel, OCT and without compound, respectively (Fig. 3). No significant difference was found between the RIN value in the Cryo-Gel and OCT embedded samples (p = 0.167). Interestingly, a significant higher RIN value was observed in the Cryo-Gel embedded samples compared to samples without compound (p = 0.003).

Skin, colon and liver tissues showed good RIN values in all different embedding compounds. Only the spleen tissue samples showed lower RIN values in all embedding compounds. In one of the skin samples without compound no RIN value could be detected while the other skin samples showed a good RIN value (Supplementary Table 1).

### DNA

**Figure 4:**
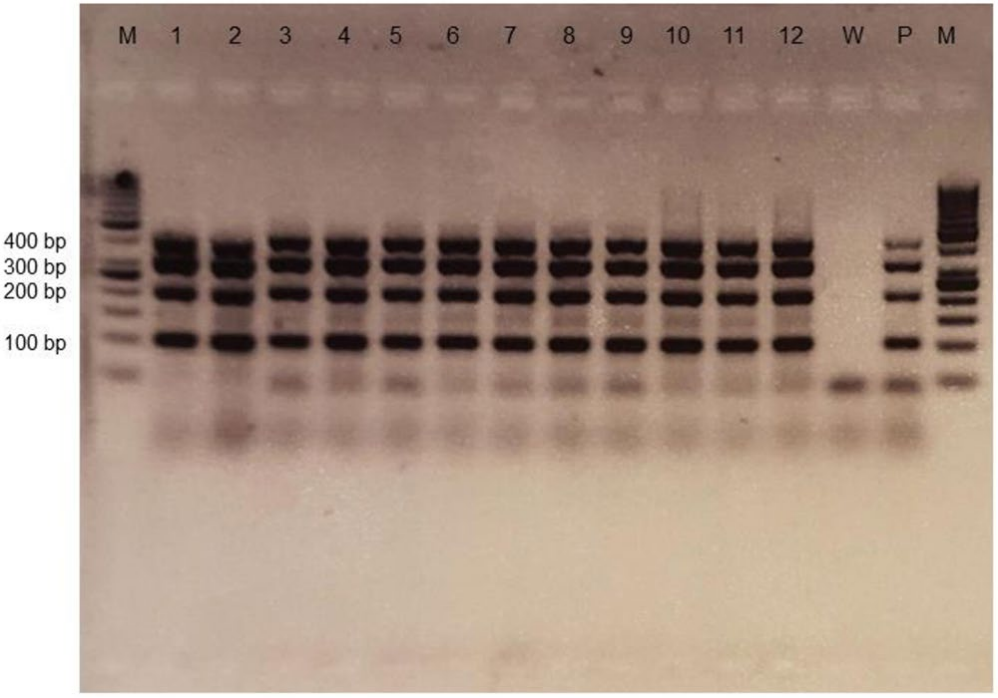
PCR results of Cryo-Gel samples (lane 1–4), OCT samples (lane 5–8) and samples without compound (lane 9–12) showing a product at 400, 300, 200 and 100 bp. All experiments included a positive control (P) and a negative water control (W).

DNA analysis using PCR was performed in duplicate on the renal samples for each embedding method. All samples showed products at 400, 300, 200 and 100 bp representing a good quality of DNA in the kidney samples (Fig. 4). Skin, colon and liver tissue samples all showed signals up to 400 bp. The spleen samples showed products up to 300 and 400 bp (Supplementary Table 2).

### Proteomics

**Figure 5:**
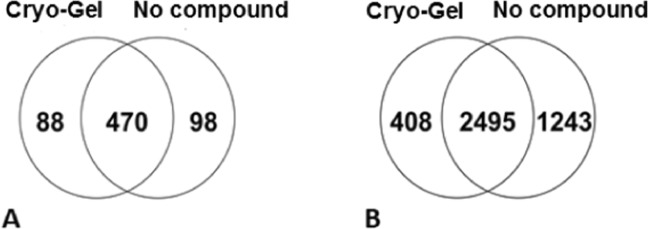
Venn diagram showing the number of different proteins (**A**) and peptides (**B**) identified by mass spectrometry and overlap between the samples embedded with Cryo-Gel and without compound.

Quantitative proteomic data from Cryo-Gel embedded renal tissues were compared to data from renal tissues without compound. The MS results showed no detectable polymers in both the Cryo-Gel samples and the samples without compound. A mean of 412 ± 64 proteins was identified in the Cryo-Gel samples and 451 ± 59 in the samples without compound (p = 0.398). In the Cryo-Gel samples 558 different proteins were identified versus 568 proteins in samples without compound. Four-hundred-seventy of the identified proteins were present in both groups (Fig. 5). A mean of 2397 ± 97 peptides was identified in the Cryo-Gel samples and 3019 ± 59 in the samples without compound (p = 0.066). In Cryo-Gel samples 2903 different peptides were observed versus 3738 peptides in samples without compound (Fig. 5).

The identified proteins were categorized by their cellular compartment and biological function using gene ontology (GO) software. Virtually identical percentages of proteins were identified in the different categories of Cryo-Gel samples and samples without compound (Supplementary Fig. 2).

**Figure 6:**
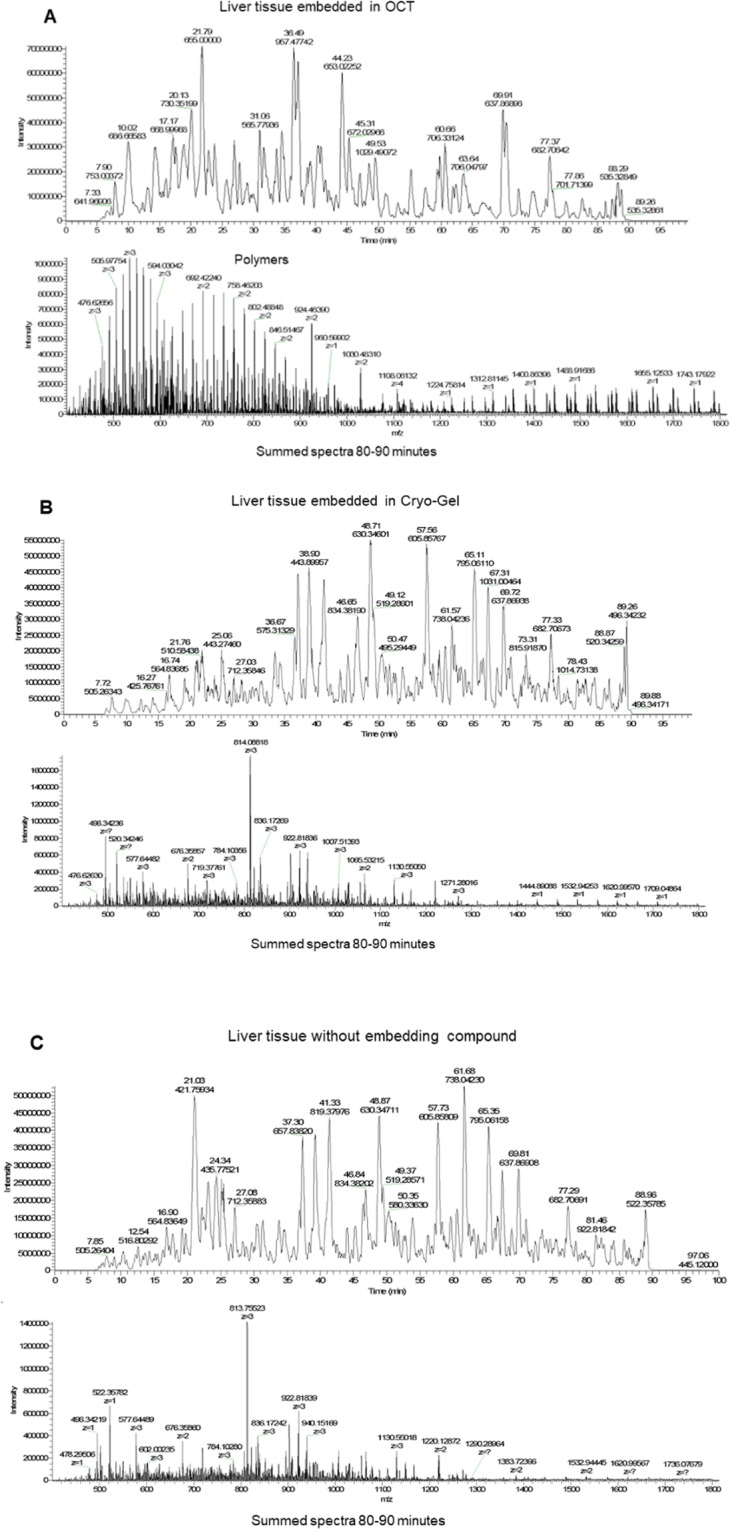
Mass spectra from liver samples embedded in OCT, Cryo-Gel and without compound. The summed spectra at 80–90 minutes show repeating polymer peaks in the OCT embedded samples disturbing the signal, but not in the samples embedded in Cryo-Gel and without compound.

In tissue samples from spleen, liver, skin and colon, no differences between protein and peptide identification were observed between the different embedding methods (Supplementary Table 3). Polymers were found in the MS results from the OCT embedded samples, but not in the Cryo-Gel embedded samples. These polymers can be observed as repeating peaks in the MS (Fig. 6) and disturb the MS signal.

### Electron microscopy

**Figure 7:**
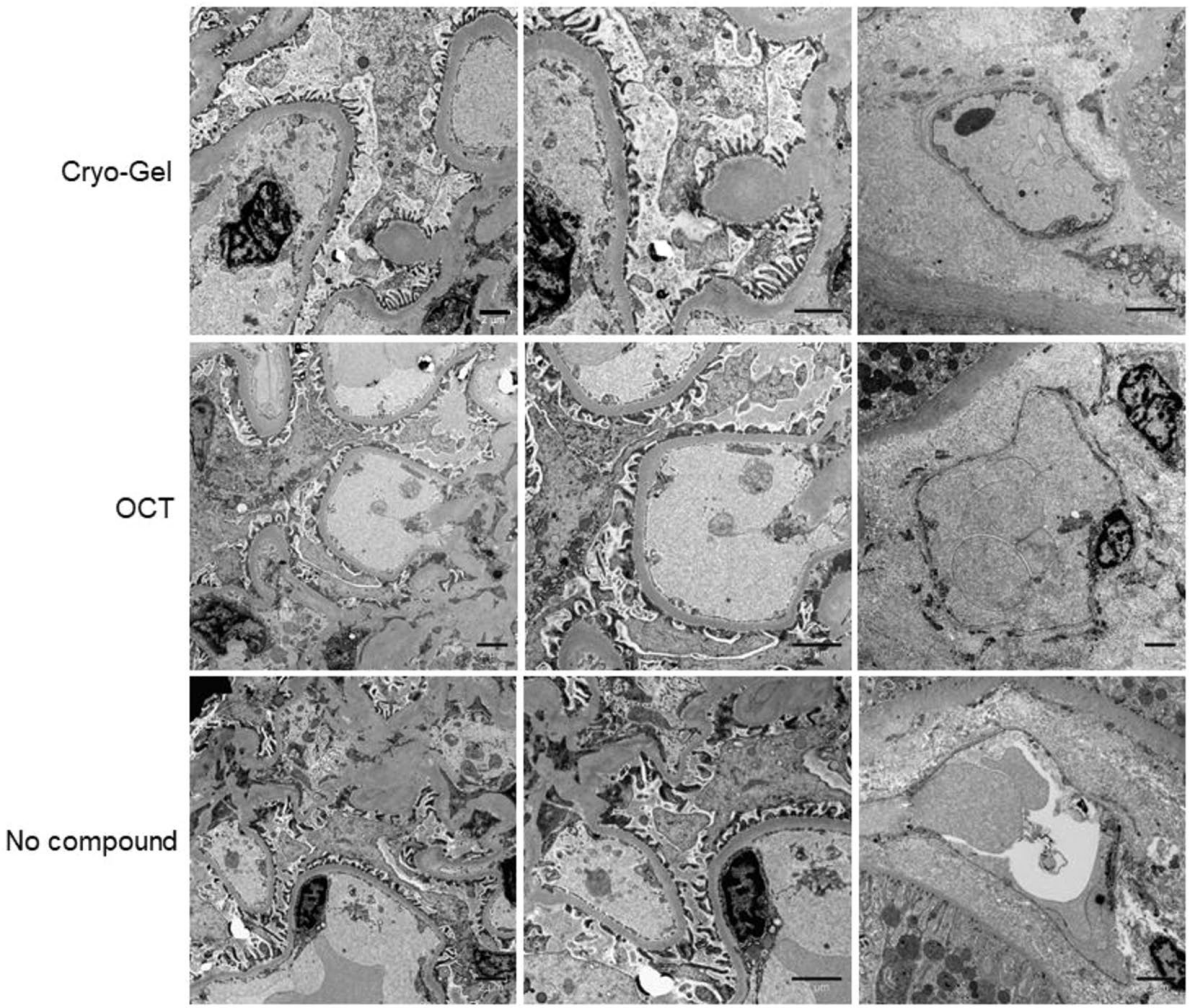
Electron microscopy for samples embedded with Cryo-Gel, OCT and without compound. Left: glomerular filtration membrane (magnification 4400x); middle: higher magnification of glomerular filtration membrane (magnification 7100x); right: peritubular capillary (magnification 7100x, 4400x and 5600x, respectively). Scale bar = 2 µm.

Electron microscopy (EM) analysis on previously snap-frozen Cryo-Gel embedded renal samples showed well-preserved material. The results were similar to OCT embedded samples and samples without tissue compound (Fig. 7). All samples showed well preserved podocytes and no depositions were observed. No differences in glomerular basement membrane thickness were found and no double contours were observed. The endothelium showed normal fenestration in all the three embedding methods.

### Immunofluorescence

**Figure 8:**
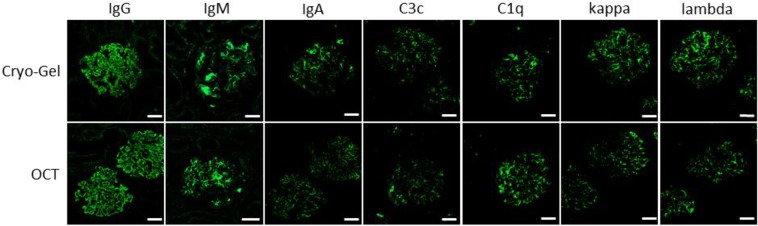
Immunofluorescence staining for IgG, IgM, IgA, C3c, C1q, kappa and lambda from renal cortex form a patient with lupus nephritis embedded in Cryo-Gel and OCT compound (magnification 20x; scale bar = 50 µm).

Immunofluorescence (IF) was performed on renal tissue from a patient with known lupus nephritis and showed similar results for Cryo-Gel and OCT embedded tissue. The intensity of the IF stainings was good in both the Cryo-Gel and OCT embedded renal tissue. No differences in staining pattern were observed between the renal samples embedded in Cryo-Gel and OCT. Intense granular membranous staining was seen for IgG. Moderate granular membranous staining was seen for IgA, IgM, C3c, C1q, kappa, and lambda (Fig. 8).

## Discussion

Adequate biobanking of renal biopsies is necessary for diagnostics and subsequent research^1^. The issues that have to be considered regarding proteomics are the quality and quantity of the proteins that can be extracted. OCT is widely used for embedding tissue for snap-freezing, however it is not ideal for MS due to interfering polymers (e.g. PVA and PEG) in the OCT^9,13^. Therefore, we investigated the use of Cryo-Gel as embedding compound. This study shows that Cryo-Gel can be used without any technical problems in MS, which is a big advantage when compared to OCT compound.

Research in kidney proteomics is a fast moving area and has mainly been focused on urine due to non-invasiveness^18–20^. However, using urine as sample source it is not possible to link the expressed proteins in kidney disease to their specific site of production and they might even originate from outside the kidney. The performance of proteomics on renal tissue itself allows for investigation of proteins within the affected kidney compartments^3^. This can be optimized using laser capture microdissection (LCM) in combination with MS. With LCM, areas of interest, such as glomeruli, interstium or tubuli can be isolated by cutting away the surrounding tissue, making it easier to evaluate protein profiles in specific structures^6,21^.

Protein extraction of samples embedded in Cryo-Gel was feasible and Cryo-Gel showed no interference in MS analysis. In renal samples, the number of proteins identified in Cryo-Gel embedded tissue was comparable to tissue without compound with a high overlap of identified proteins, although there were slightly fewer peptides identified (NS).

MS on OCT embedded tissue was performed on spleen, skin, colon and liver samples. In these tissue samples, the number of proteins detected was not significant different between the Cryo-Gel and OCT embedded tissues and the tissues without compound. Although, polymers were detected in the MS data from the OCT embedded samples and not in the Cryo-Gel embedded samples. The polymers in the OCT embedded tissue suppress other signals in the MS^9,13^. In the present study, the MS data is obtained on an older type of mass spectrometer which is slower compared to the newest generation of MS equipment. The polymers mainly suppress lower abundant signals in the samples which are, because of the speed of the used instrument, probably not sequenced. This could be a potential explanation for the fact that no difference in the amount of protein identifications is observed even while the polymer signal is clearly present in the acquired spectra.

The polymers in OCT are very hydrophobic and have a strong interaction with the C18 stationary phase of the MS columns. Stringent washing steps of the columns are required to remove these polymers from the columns. A portion of the polymers may even bind irreversibly and effect the lifetime and the separation performance of the columns considerably. The performance of MS analysis on OCT embedded tissue samples is therefore discouraged. We recommend the use of Cryo-Gel embedding compound to not exclude the possibility of MS analysis.

Methods to remove OCT from the tissue samples before protein extraction and MS have been described^10,22^. Recently, Vrana *et al*. described an optimized method to extract proteins from OCT embedded tissue samples^12^. They removed the excess of frozen OCT compound around the kidney tissue samples before thawing the tissue. Then diethyl ether/methanol extraction was performed to remove the remaining of OCT. To decrease the risk of ion suppression by OCT in the MS analysis, a second cleaning step with methanol/chloroform/water extraction was added to the procedure. This shows that the removal of OCT from the tissue samples for MS analysis is a time-consuming procedure which takes multiple steps.

Unfortunately, the chemical compounds of Cryo-Gel are confidential. Since Cryo-Gel did not cause interference in MS analysis, we assume that Cryo-Gel does not contain polymers like those in OCT compound. It is also possible that Cryo-Gel only contains polymers that can be broken down in the preparation steps for proteomic analysis (trypsinization and digestion) and that therefore no problems in MS analysis are observed.

Tissue morphology and different stainings on the Cryo-Gel embedded samples showed similar results to tissue embedded in OCT and those without compound. Also, the quality of DNA was good in the different tissues and no major differences were observed between the different embedding compounds. RNA showed comparable yield in Cryo-Gel and OCT embedded tissues and tissues without embedding compound. No significant difference was observed between the RIN value in Cryo-Gel and OCT embedded renal tissues. Interestingly, renal tissue without embedding compound had a significantly lower RIN value, although the RNA was still of good quality. Spleen samples showed a lower RIN value in all different embedding methods compared to the other tissue types. This is probably caused by external conditions, such as changes in tissue temperature, causing RNA degradation, for which spleen tissue is more sensitive than the other tissues that were used.

A limitation of the present study is the small number of tissue samples used for evaluation. However, different types of tissue were analyzed and different experiments to test the use of Cryo-Gel were performed. Furthermore, two samples of all different tissue types were obtained for each embedding compound and the experiments on the renal tissue samples were performed in duplicate.

A vital issue for tissue biobanking for future research is the maintaining of protein stability over time. We show that Cryo-Gel compound has good protein preservation in renal tissue after 9 months of storage at −80 °C. Further research is necessary to analyze the protein stability of Cryo-Gel embedded tissue after a longer storage time.

We conclude that Cryo-Gel is an excellent compound for snap-freezing renal biopsies for both diagnostic and research purposes involving not only histomorphology and immunofluorescence, but also RNA, DNA and protein analysis.

## Methods

Fresh samples of normal renal cortex were obtained within 2 hours after surgery from a 46-year-old male who had a total kidney resection due to a renal malignancy. Samples were snap frozen using either Cryo-Gel embedding medium (Leica, Surgipath, the Netherlands), Tissue-Tek OCT Compound (Sakura Finetek, the Netherlands) or no embedding compound. The quality of tissue histology, EM, DNA, RNA and proteins was evaluated (Fig. 1). In addition, fresh samples from normal spleen, skin, liver and colon from surgical resections due to malignancies were collected. These tissues were snap frozen using Cryo-Gel, OCT and no compound. The quality of tissue histology, DNA, RNA and proteins was evaluated in these tissues. For each medium two samples were embedded. For renal tissues, the experiments were performed in duplicate. In addition, a section of a renal biopsy from a patient with known lupus nephritis was used to evaluate the quality of IF on Cryo-Gel embedded tissue.

### Cryosectioning

Tissue samples were placed in a cryomold and covered with either Cryo-Gel, OCT or no compound and immersed in cold isopentane with liquid nitrogen until snap-frozen. After solidification, the cryomolds were removed and tissues were stored at −80 °C until cryosectioning. Routine cryosectioning was performed using a cryostat (Leica CM 1950, Netherlands) at −20 °C. For the renal tissues, the experiments were performed after 9 months of storage. For all other tissue samples, the experiments were performed within a week after snap-freezing. For those tissues frozen without embedding compound, a small drop of NaCl was used to mount the tissue sample on the cryostat microtome object holder to keep the tissue in place. Four micrometer cryostat sections were cut from the samples frozen with Cryo-Gel, OCT and without compound in a cryostat (Leica CM 1950, Netherlands) and subsequently stained with H&E and evaluated by light microscopy.

### RNA

Five 10 μm cryostat sections from the tissue samples frozen with Cryo-Gel, OCT and without embedding compound were cut to analyze the quality and quantity of the RNA. The cryostat sections were placed in 700 µL Qiazol (Qiagen, Germany). The tissue sections were disrupted by shaking the tubes vigorously for about 5 sec. Total RNA was isolated according to the protocol of the miRNeasy mini kit (Qiagen, Germany). RNA samples were subsequently placed on ice for approximately 30 min prior to RIN measurement with the Bioanalyzer (Agilent RNA 6000 Nano kit, Agilent Technologies, Germany)^23^. A RIN value of ≥6.5 was used as a cut-off for good RNA quality^24,25^.

### DNA

Five cryostat sections of 10 µm from the tissue samples frozen with Cryo-Gel, OCT and without embedding compound were cut and placed into individual eppendorf vials. Proteinase K (7.5 AU, Qiagen, Germany) was added to all the samples and left for protein digestion at 56 °C. Then, the samples were placed at 95 °C for 10 minutes for proteinase K deactivation and spun down at 14,000 RPM for 5 minutes. For subsequent DNA quality assessment, we applied multiplex PCR on isolated DNA from the samples embedded in Cryo-Gel, OCT and without compound. PCR was performed using the following primers: TBXAS1//X9U, forward PCR, GCC CGA CAT TCT GCA AGT CC; reversed PCR, GGT GTT GCC GGG AAG GGT T (PCR product of 100 bp); RAG1/X2U, forward PCR, TGT TGA CTC GAT CCA CCC CA; reversed PCR, TGA GCT GCA AGT TTG GCT GAA (PCR product of 200 bp); PLZF/X1U, forward PCR, TCG GAT GTG GTC ATC ATG GTG; reversed PCR, CGT GTC ATT GTC GTC TGA GGC (PCR product of 300 bp) and AF4/X11U, forward PCR CCG CAG CAA GCA ACG AAC C and reversed PCR GCT TTC CTC TGG CGG CTC C (PCR product of 400 bp). PCR was performed in 15 μl reaction volumes containing 4.5 μl H_2_O, 7.5 μl KAPA2G HotstartReadymix, 1 μl each of forward and reverse primer with concentration of 100 ng/μl and 1 μl DNA. Cycling conditions were 3 min at 95 °C for initial denaturation, 35 cycles each with 15 sec at 95 °C, 15 sec at 60 °C and 15 sec at 72 °C for denaturation, annealing and extension, and a final elongation step of 7 min at 72 °C. This was followed by electrophoresis on agarose gel (1%) in Tris Broom EDTA (TBE) to analyze the bands of the corresponding PCR products.

### Proteomics

In the renal samples, frozen tissue in Cryo-Gel and without compound was processed for proteomic analyses. In the other tissue samples (spleen, skin, liver and colon), proteomics was performed on the Cryo-Gel embedded samples, samples without compound and the OCT embedded tissue. Ten cryostat sections of 5 µm from the tissue samples were processed into 100 μl digestion buffer containing 0.1% Rapigest (Waters, United States) in 50 mM ammonium bicarbonate (Sigma Aldrich, Netherlands) and was lysed through sonication at 70% amplitude. Proteins were incubated at 60 °C for 30 minutes after reducing with a 100 mM dithiothreithol solution (Sigma Aldrich, Netherlands), and alkylated with a 300 mM iodoacetamide solution (Sigma Aldrich, Netherlands) at room temperature and in darkness for 30 min. Then trypsin (600 ng/sample, Promega) was added and samples were digested overnight at 37 °C. The digested samples were then acidified with trifluoroacetic acid (Sigma Aldrich, Netherlands) to a final concentration of 0.5%, and spun down at 14,000 RPM for 30 minutes. Supernatants were collected and transferred to a LC glass vial for further LC-MS measurement. Digested proteins were separated on a nano-LC (Ultimate 3000, Thermo Fisher Scientific, Germany) equipped with a reverse phase analytical column (PepMap C18, 75 μm ID 15 cm, 3 μm particle size and 100 Å pore size, Thermo Fisher Scientific, Netherlands) and peptides were eluted with the following gradient of solvent A and B: 4% to 38% of solvent B in 90 minutes. Solvent A consists of 0.1% aqueous formic acid in water and solvent B consists of 80% acetonitrile and 0.08% aqueous formic acid.

MS measurements were performed on a LTQ Orbitrap XL. For electro-spray ionization (ESI), nano ESI emitters (New Objective, United States) were used and a spray voltage of 1.5 kV was applied. For MS detection a data-dependent acquisition method was used: a high resolution MS scan from 400 to 1600 Th was performed in the Orbitrap, resolution 30.000, lock mass was set to 445.120025 Th (protonated (Si(CH3)2 O)6). Based on this MS scan, most intense ions were consecutively isolated and fragmented with a ‘Top five’ setting. After selection ions were placed on a dynamic exclusion list for 3 min. Settings for MS/MS fragmentation were: 30 ms maximal fill time; isolation width of 2.0 *m/z*; fragmentation by collision induced dissociation (CID) applying 35% normalized collision energy; detection of fragment ions in the linear ion trap. After precursors were selected for MS/MS, they were excluded within a tolerance range of 10 ppm for further MS/MS analysis for 1 min.

To calculate the number of identified proteins and peptides we used Scaffold (version 4.8.4, Proteome Software Inc., United States). Filter criteria for protein identification were set to >99% protein probability and >99% peptide probability. Proteins that were associated with ≥1 peptides were included. The mean number of proteins identified in samples embedded in Cryo-Gel, OCT and without compound were calculated. Furthermore, the sum of all different proteins in each category was calculated in the renal samples.

### Formalin-fixed paraffin embedded

Half of each snap-frozen tissue sample embedded in Cryo-Gel, OCT and without embedding compound was subsequently processed for FFPE. Frozen tissue samples were thawed in formalin and after fixation, the tissue was dehydrated, cleared and infiltrated with melted paraffin wax. After solidification, 4 µm sections were cut and stained according to routine diagnostic practice for H&E. For the renal tissue, 2 µm sections were cut and subsequently stained by H&E, periodic acid–Schiff (PAS), Jones and Trichrome. In addition, immunohistochemical stainings for AE1/AE3 and CD31 were performed on the renal tissue to asses if snap freezing in different medium effects immunohistochemical staining. On the liver samples, PAS, Trichrome, Sirius Red staining and Iron staining were performed. The staining protocols are described in the Supplementary Methods. All slides were microscopically evaluated by three pathologists (MS, MD and MCvG). The quality of the different stainings was assessed by comparing the intensity of staining in both the cytoplasm and nuclei. Also, the degree of background staining was compared. The OCT embedded samples were used as golden standard.

### Electron microscopy

The remaining tissue of snap-frozen kidney samples were processed for transmission EM. Semi-thin (1 μm) survey sections were stained with toluidine blue and investigated for the presence of glomeruli. Ultra-thin (40 nm–60 nm) sections were cut, mounted on copper grids and contrasted with uranyl acetate and lead citrate. The sections were examined using a Morgagni 268D EM microscope (Thermo Fisher Scientific, United States). EM quality was assessed by two pathologists (MS and MCvG) by evaluating different structures; glomerular basement membrane (e.g. thickness, double contours, splitting), podocytes (effacement of podocyte foot processes), endothelium (swelling, loss of fenestration, tubuloreticular inclusions) and depositions (subepithelial, intramembranous, subendothelial, mesangial).

### Immunofluorescence

To determine the effects of Cryo-Gel on IF, a biopsy sample of cortical renal tissue from a patient with known lupus nephritis was split. One half was embedded in OCT and the other half in Cryo-Gel. Three micrometer sections were cut and IF was performed for IgG, IgA, IgM, C3c, C1q, kappa and lambda according to standard diagnostic routine for both embedding compounds. The quality of the different stainings was assessed by two pathologists (MS and MCvG) by comparing the structures were deposition was observed, the pattern of staining (linear, granular) and the intensity of staining. The OCT embedded sample was used as golden standard.

### Statistical analysis

SPSS Statistics version 24.0 was used (IBM SPSS Statistics for Windows. Armonk, NY:IBM Corp.). The One-way Anova was used to compare RIN values, RNA concentration and the number of proteins and peptides between the different embedding compounds. Differences with a (two sided) p-value ≤ 0.05 were considered statistically significant.

### Medical ethics

The study protocol was consistent with international ethical and professional guidelines. The use of anonymized rest material is regulated under the code for proper secondary use of human tissue in the Netherlands. The local medical ethics committee of the Erasmus MC, University Medical Center Rotterdam, Rotterdam, The Netherlands, approved that our study is exempt from the requirement for approval (MEC-2016-350). Informed consent to the use of medical record data and rest material was waived in accordance with the Dutch regulations.

## Supplementary information

Supplementary File
