# Supplementary material for: Cryo-Gel embedding compound for renal biopsy biobanking
Source: Sci Rep. 2019 Oct 24;9:15250. doi: 10.1038/s41598-019-51962-8 (PMC6813323; doi:10.1038/s41598-019-51962-8)
Supplement: Supplementary file 1 — Supplementary File [file 41598_2019_51962_MOESM1_ESM.pdf]

Cryo-Gel embedding compound for renal biopsy biobanking

Malou L.H. Snijders[1*], Marina Zajec[2, 3], Laurens A.J. Walter[1], Remco M.A.A. de Louw[1], Monique H.A Oomen[1], Shazia Arshad[1], Thierry P.P van den Bosch[1], Lennard J.M. Dekker[2], Michail Doukas[1], Theo M. Luider[2], Peter H.J. Riegman[1], Folkert J. van Kemenade[1] and Marian C. Clahsen-van Groningen[1]

[1]*Department of Pathology, Erasmus MC, Rotterdam;*

[2]*Department of Neurology, Erasmus MC, Rotterdam;*

[3]*Department of Clinical Chemistry, Erasmus MC, Rotterdam.*

Supplementary Table 1. RNA analysis in spleen, skin, liver and colon tissue.

	Embedding compound		RIN value	RNA concentration (ng/µl)
Spleen	OCT	1	5,4	90
		2	5,5	119
	Cryo-Gel	1	5,1	297
		2	5,1	357
	No compound	1	5,4	163
		2	5,3	259
Skin	OCT	1	8,5	11
		2	9,9	51
	Cryo-Gel	1	9,6	90
		2	9,2	17
	No compound	1	8	22
		2	NA	3
Liver	OCT	1	8,9	47
		2	8,6	24
	Cryo-Gel	1	9	200
		2	8,9	91
	No compound	1	8,8	262
		2	8,9	111
Colon	OCT	1	8,5	14
		2	8,7	21
	Cryo-Gel	1	7,1	10
		2	10	26
	No compound	1	8	17
		2	9,7	10

The RIN (RNA Integrity Number) and RNA concentration were measured in the spleen, skin, liver and colon tissue samples embedded in OCT, Cryo-Gel and without compound.

NA = not available

Supplementary Table 2. DNA analysis in spleen, skin, liver and colon tissue.

		OCT		Cryo-Gel		No compound	
		DNA concentration (ng/ul)	PCR	DNA concentration (ng/ul)	PCR	DNA concentration (ng/ul)	PCR
Spleen	1	33,1	signal up to 400bp	26,0	signal up to 300bp	42,3	signal up to 400bp
	2	35,7	signal up to 400bp	54,0	signal up to 300bp	36,2	signal up to 300bp
Skin	1	2,52	signal up to 400bp	2,97	signal up to 400bp	3,58	signal up to 400bp
	2	0,99	signal up to 400bp	1,50	signal up to 400bp	1,12	signal up to 400bp
Liver	1	10,5	signal up to 400bp	16,5	signal up to 400bp	17,1	signal up to 400bp
	2	9,4	signal up to 400bp	15,7	signal up to 400bp	14,0	signal up to 400bp
Colon	1	3,91	signal up to 400bp	3,23	signal up to 400bp	3,93	signal up to 400bp
	2	4,69	signal up to 400bp	3,12	signal up to 400bp	3,16	signal up to 400bp

PCR was performed on the spleen, skin, liver and colon tissue samples embedded in OCT, Cryo-Gel and without compound. DNA concentration and PCR signals were measured.

Supplementary Table 3. Proteomic analysis in spleen, skin, liver and colon tissue.

	Embedding compound		Protein identification	p-value	Peptide identification	p-value
Spleen	OCT	1	367	0.064	2214	0.187
		2	370		2389	
	Cryo-Gel	1	388		2484	
		2	409		2655	
	No compound	1	370		2279	
		2	366		2419	
Skin	OCT	1	73	0.866	678	0.683
		2	64		562	
	Cryo-Gel	1	63		555	
		2	69		595	
	No compound	1	52		467	
		2	74		623	
Liver	OCT	1	463	0.145	3251	0.455
		2	473		3285	
	Cryo-Gel	1	474		3304	
		2	473		3285	
	No compound	1	463		3259	
		2	460		3287	
Colon	OCT	1	291	0.518	2422	0.343
		2	144		1321	
	Cryo-Gel	1	246		1460	
		2	124		553	
	No compound	1	252		1779	
		2	329		2197	

Proteomic analysis was performed on the spleen, skin, liver and colon tissue samples embedded in OCT, Cryo-Gel and without compound. The number of proteins and peptides identified in samples embedded in OCT, Cryo-Gel and without compound showed no significant differences.

Supplementary Figure 1.

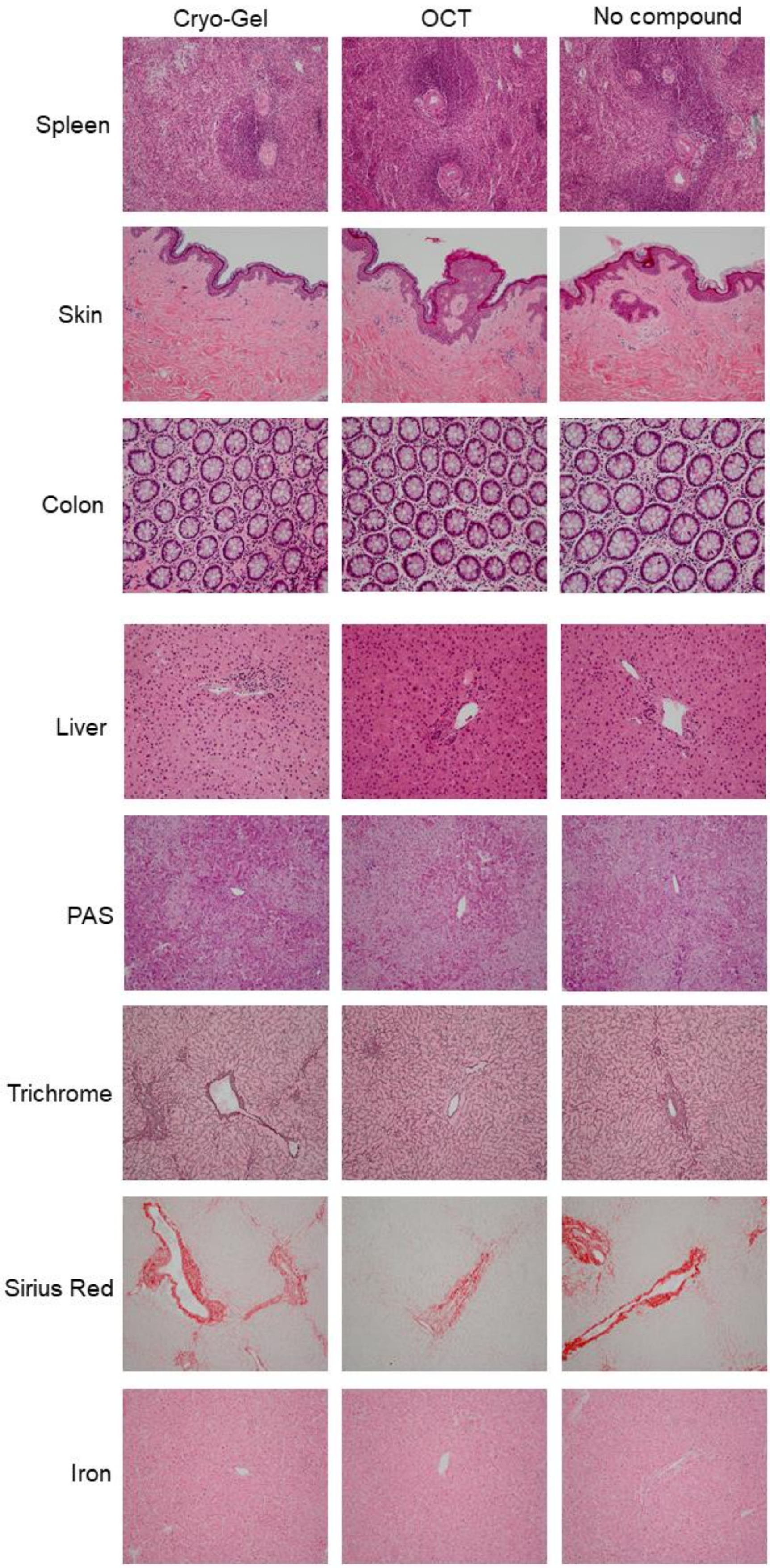

H&E staining on spleen, skin and colon samples and H&E, PAS, Trichrome, Sirius Red and Iron staining on liver samples embedded in Cryo-Gel, OCT and without compound converted to FFPE material (magnification 20x).

Supplementary Figure 2.

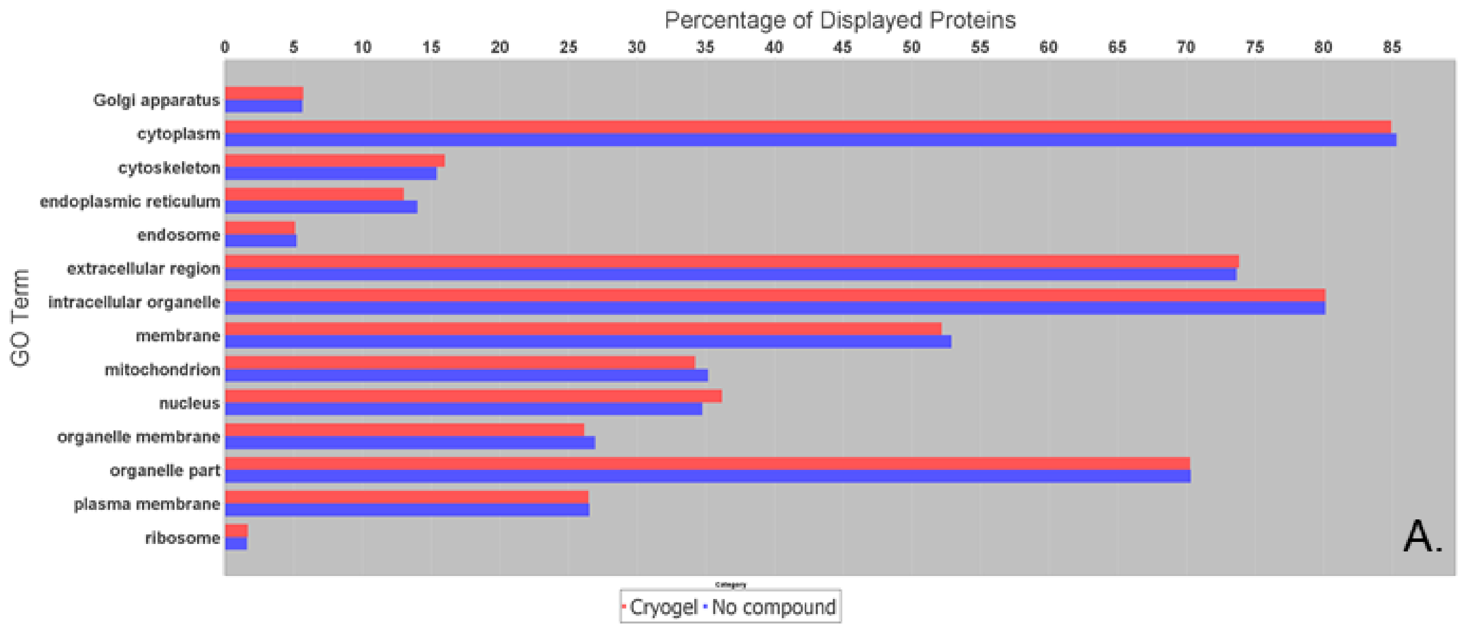

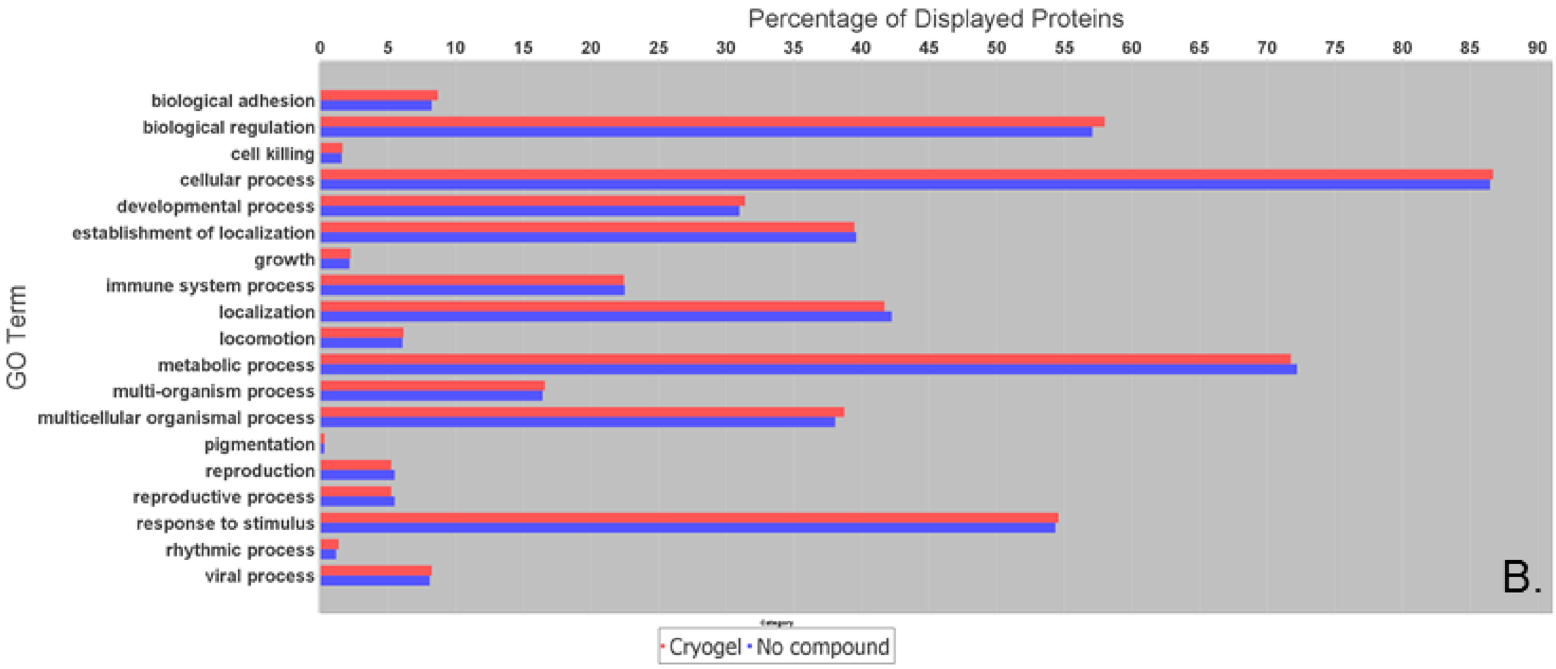

Gene ontology analysis of proteins identified by mass spectrometry for renal samples embedded in Cryo-Gel and without compound categorized by cellular localization (A) and biological process (B).

Supplementary Methods.

Protocols of different stainings on FFPE sections:

FFPE sections were stained with H&E using the Automated Ventana Symphony stainer according to manufactures instructions. Histochemical staining for PAS, Trichrome, Jones, Sirius Red and Iron was performed were performed on automated Ventana special stains after deparaffination.

In brief for PAS, slides were heated till 75˚C and diastase was added for 12 minutes. Next, Schiffs reagents was added for 20 minutes and lastly a counterstain with hematoxylin was performed.

For Jones, slides were heated till 75˚C and then cooled till 60 ˚C. Jones Silver B reagents was added for 16 minutes and lastly eosin was added as counterstain.

For Trichrome, Trich bouins A was added for 32 minutes and after additional washing steps, hematoxylin A+B was added for 12 minutes. After further washing steps, Trich Red was added for 8 minutes, Trich Mordant was added for 12 minutes and Trich Blue was added for 16 minutes.

For Sirius Red, Fosformolybdeen acid 0,2% was added for 2 minutes and after a washing step, Picro-Sirius Red was added for 60 minutes.

For the Iron staining, slides were heated till 75˚C and Iron reagent A was added for 4 minutes. After a washing step, Iron reagent B was added for 4 minutes. After a washing step, Iron NFR was added for 8 minutes.

Immunohistochemical staining for AE1/AE3 and CD31 was performed. For CD31, following deparaffinization and heat-induced antigen retrieval with CC1 (#950-124, Ventana) for 16 minutes, the tissue samples were incubated with CD31 (JC70, #760-4378, Cell Marque) for 20 minutes at 36˚C.

For AE1/AE3, following deparaffinization, protease for 3-4 minutes (protease3: 7601-2020, Ventana) and heat-induced antigen retrieval with CC1 (#950-124, Ventana) for 20 minutes, the tissue samples were incubated with AE1/AE3 (E08171, #760-2135, Ventana) for 4 minutes at 37˚C.

All stainings were done with appropriate controls.
